# Choosing the perfect shot – The loaded narrative of imagery in online news coverage of vaccines

**DOI:** 10.1371/journal.pone.0199870

**Published:** 2018-06-27

**Authors:** Andrew G. Wu, Ashish S. Shah, Tara S. Haelle, Scott A. Lunos, Michael B. Pitt

**Affiliations:** 1 Department of Pediatrics, University of Minnesota Masonic Children’s Hospital, Minneapolis, Minnesota, United States of America; 2 Core Topic Leader of Medical Studies, "Covering Health", Columbia, Missouri, United States of America; 3 Biostatistical Design and Analysis Center, University of Minnesota, Minneapolis, Minnesota, United States of America; The University of Warwick, UNITED KINGDOM

## Abstract

Images in health communication have been shown to affect perspectives and attitudes towards health issues including vaccination. We seek to quantify the frequency of images used in online news coverage of vaccines that may convey varying sentiments about vaccination. To capture a breadth of vaccine-related news coverage, including international sources, we searched the following terms in Google News Archives: “autism and vaccine”, “flu and vaccine”, and “measles and Disneyland”. We developed a coding tool that classified images as negative (eg, screaming child), positive (eg, happy child), neutral (eg, vaccine vial), or irrelevant (eg, picture of journalist). All images were coded independently by two researchers and discussed for consensus. We analyzed 734 images. Of the images which featured vaccines and/or a medical encounter (322), 28% had negative features and 30% had positive features. The remaining 137 images (43%) were neutral. There was no statistically significant difference between proportions of negative and positive imagery for each pair of search terms, which may be a reflection of random image selection. Ultimately, nearly one in eight images included in vaccine-related news coverage contains negative features which may be selected without careful consideration of the potential negative impact on public health initiatives regarding vaccination.

## Introduction

Vaccine hesitancy is prevalent [1–3]. Reasons for declines in adherence to the recommended vaccine schedule are complex and include parental concerns that vaccines are not safe or necessary [1, 4]. These perceptions can be influenced by misinformation in public health communication [3]. The rise of vaccine hesitancy has occurred as the public is increasingly turning to online search engines to inform their healthcare decisions [5–8]. Online news media, which is often discovered via these searches, is cited as a trusted source for obtaining accurate health information when making these healthcare decisions [9–12].

In addition to the messages conveyed in the written text of a news article, the images chosen to accompany the text can shape the public’s reaction to the message [13, 14]. In contrast to text, images have a distinct emotive impact that can be readily absorbed in an unmediated manner without reflection and often serve to verify the authenticity of the text by providing a visual “reality” to the story [14]. Psychological literature has posited that various pathways of social cognition are employed when visual material is used in communication. For example, specific emotive pathways such as disgust- and fear-based stimuli have been suggested to play a large part in visual communication. The concept of “magical contagion” plays a role here, where, for example, a clearly distressed child receiving a needle injection can invoke both a visceral feeling of disgust and a level of magical thinking [15]. The idea or visualization of a needle and a crying child then becomes contaminated or offensive. Another is the availability heuristic, which teaches that an event is deemed more likely to occur the more readily it can be recalled [16]. These mechanisms can allow visual material to sway public opinion in a way that text often cannot, which can make their use in public health communication so powerful.

Historically, images have played an important role in promoting public health initiatives around vaccination. Images of Elvis Presley receiving the polio vaccine with a smile during a taping of the *Ed Sullivan Show* or those depicting the long lines of people waiting for their smallpox vaccine were heavily used to promote vaccination [17]. Conversely, images may also undermine public health initiatives, intentionally or unintentionally. For example, repeated exposure to images such as infants depicted sleeping in prone position or children riding bicycles without helmets in parenting magazines can serve to normalize or even subtly endorse these behaviors even when that is clearly not the intent [18–20].

The images chosen by writers and editors to accompany vaccine news coverage have the potential to subtly convey positive or negative sentiments about vaccines which may support or undermine these public health initiatives. For example, when these pictures contain screaming children or long needles as the focal point, it is possible that the anxiety evoked by the pictures may counteract the content of the message in the article.

In our study, we aimed to determine the frequency of images accompanying vaccine news coverage in online media which contain features that may promote positive or negative aspects about vaccines or vaccination, and we were able to achieve this aim. By developing a coding tool and quantifying the prevalence of these images, we hope to inform writers and editors of vaccine-related news stories of the importance of carefully selecting images accompanying their articles.

## Materials and methods

We searched the “News Archives” section of Google–the most frequently used internet search engine at the time of our study [21]–using the Incognito feature of the Google Chrome browser, which eliminates personal search history, location, and cookies from informing search results. In order to capture a range of types of news coverage, we selected search terms that spanned various types of vaccine stories. We chose the search terms: “autism and vaccine” with the hopes of capturing news coverage of the anti-vaccine movement [22, 23]; “flu and vaccine” to capture a periodic news cycle linked to a public health initiative aimed for both children and adults [24, 25]; and “measles and Disneyland” in order to capture coverage of a single event (the measles outbreak at Disneyland in 2015) related to consequences of under-vaccination. Our goal was not to be exhaustive in this search, but rather to capture stories which cover a spectrum of opinions about vaccines in recent news. As these three searches address topics including outbreaks, public health initiatives, and vaccine hesitancy, we hoped this may be an avenue to generate a sample of photos within news coverage from which clear trends of photo selection could be made, and more importantly could be used to highlight a framework for future selection of photos by key stakeholders Google News Archives covers international sources, though our search terms relegated most of the news to North American sources. Searches were performed during June 17–22, 2016 from Minneapolis, MN.

For each search term, we reviewed all articles on the first ten search pages sorted by relevance and downloaded all images that accompanied the article. Images displayed at the start of a video were included if that video was primed with the image serving as a still frame within the video player. Images on the article’s webpage that were not part of the article itself were excluded (eg, images linking the reader to a related article or advertisements were not included in the analysis).

Using multiple iterations of a modified Delphi process carried out via teleconferences and email, we developed a coding tool that classifies features found in vaccine imagery as: *negative* (visibly distressed child and/or parent, needle as the focal point by taking up 50% or more of the image, multiple needles); *positive* (visually happy or calm child and/or parent, depictions of a conversation with health provider); *neutral* (vaccine supplies only or vaccine being administered without any faces depicted); or *irrelevant* if they did not contain any imagery directly related to vaccines or vaccine administration (eg. picture of Disneyland, picture of reporter) (Table 1). Images were coded as negative if at least one negative feature was present even if positive features were also present (eg, a screaming child with smiling parents was coded as possibly promoting negative sentiments). As we were particularly concerned with the framework established in the social psychological literature which describes emotion as the main mechanism through which imagery affects its audience, only sentiment presented visually (i.e. in the photographs) was codified and measured in this study.

**Table 1:** Criteria used to code images accompanying online vaccine news coverage with example pictures.

Classification	Image Characteristics	Links to Representative Pictures from Dataset
Negative*		
*May promote negative sentiments about vaccination*	At least one of the following: - Visibly distressed child/parent (crying, screaming, wincing) - Needle taking up 50% or more of image - Needle is focal point of image - Multiple needles	Screaming Child: bit.ly/2HzfOUT Screaming Child: bit.ly/2r0zv1F Needle Takes Up 50%: bit.ly/2Hv86Lr Multiple Needles: bit.ly/2HYGMJ0
**Positive**		
*May promote positive sentiments about vaccination*	No negative features AND at least one of the following: - Visually happy/calm child and/or parent and/or provider - Depiction of dialogue with health provider	Smiling During Vaccination: bit.ly/2Jwse0D Smiling Post Vaccination: bit.ly/2qZzGdU Calm Child: bit.ly/2HTuNN2
**Neutral**		
	No negative of positive features AND at least one of the following: - Vaccine administered with no faces depicted - Vaccine supplies	Administration: bit.ly/2I56qK0 Supplies: bit.ly/2HxsT0Y
**Irrelevant**		
	Any image that does not fall in other categories	DNA: bit.ly/2HRT328

*Any negative feature classified an image as “negative” despite the presence of other features

Of note, our definition of a negative image included images showing children in distress, which included those suffering from a vaccine-preventable disease. Though prior studies have shown that images can impact perspectives and attitudes regarding health issues including vaccines [13, 14, 26, 27], it remains unclear if showing images of children suffering from the consequences of not being vaccinated (eg, a coughing child with pertussis, or a child with the rash of measles) increases or decreases intention to vaccinate. Though a previous study [27] has suggested that images of sick children increase expressed beliefs in a link between the MMR vaccine and autism, recent studies [26] have been unable to replicate this finding. Of note, this coding ultimately applied to only one image in our study–a child with measles–and so its coding as negative had little effect on our results.

Two authors coded the images independently. We assessed inter-rater reliability of the initial coding using Cohen’s Kappa statistic comparing both authors’ coding of all the images. For the final data set, the two authors met and compared their independent coding for each image, discussing areas of disagreement until consensus was reached [28]. Though coders were aware of the hypothesis, experimenter bias was minimized by development and finalizing of the coding criteria by all investigators (including those who did not collect data) prior to data collection and agreement to follow the coding criteria literally despite possible disagreements with what the image is “meant” to portray. We recorded absolute numbers and percentages for the image coding classification, using chi-square tests to assess associations between the classification and the three pairs of search terms. P-values for pairwise comparisons were adjusted for multiple comparisons using a Bonferroni method. A p-value less than 0.05 was deemed statistically significant. SAS V9.3 (SAS Institute Inc., Cary, NC) was used for the analysis.

## Results

A total of 734 images accompanying the news media were obtained across all search terms with a similar number of pictures obtained per search term (230 images for the autism/vaccine search (31.3%), 232 for flu/vaccine search (31.6%), and 272 for the measles/Disneyland search (37.1%). Of the total 734 images, 36 were duplicates. Cohen’s kappa statistic for the initial coding classification between the two coders was 0.77 (95% CI 0.74–0.80), consistent with a “substantial agreement” between the coding [29].

Table 2 provides a breakdown of the classification of the images for each search term. The majority of the images used were coded as *irrelevant* (56.1%; 412) as they did not feature visuals pertaining to vaccination itself. 18.7% (137) of the images were coded as *neutral*; 13.1% (96) were coded as *positive*; and 12.1% (89) of the images were coded as “negative.” After removing the irrelevant images from the analysis, 42.5% were neutral, 29.8% contained positive features, and 27.6% contained negative features. Table 1 contains examples of images from our search for each coding scheme.

**Table 2:** Descriptive statistics of outcomes by search terms.

Search Terms				
Image Classification	Autism + Vaccine	Flu + Vaccine	Measles + Disneyland	Total
Positive, n (% of search)	34 (15)	44 (19)	18 (7)	96 (13)
Negative	29 (13)	36 (16)	24 (9)	89 (12)
Neutral	33 (14)	56 (24)	48 (18)	137 (19)
Irrelevant	134 (58)	96 (41)	182 (67)	412 (56)
**Total, n (% overall)**	**230 (31)**	**232 (32)**	**272 (37)**	**734**
**Pairwise Comparison:** autism + vaccine vs. flu + vaccine: p = 0.008 autism + vaccines vs. measles + Disneyland: p = 0.02 flu + vaccines vs. measles + Disneyland: p = <0.0001				

Data represent raw count and proportions of the total images per search term. Pairwise comparisons attempted to detect a difference between the proportions of all connotations between two given searches. Statistical significance was defined as p<0.05.

Including images coded as *irrelevant*, there was statistically significant variation of the types of images used across the three different search terms (p < .05) (Table 2). The images derived from the measles/Disneyland search were the least likely to have features that may have either promoted or undermined vaccination as the proportion of positive (7% of this search) and negative (9%) images were the lowest among the three searches. Conversely, the images yielded by the flu/vaccine search had the highest proportion of images identified as having either positive (19%) or negative (16%) features. When considering only pictures which had a potential vaccine bias (i.e. coded as negative or positive), there was no statistically significant difference between the proportions of negative and positive imagery for each search term, with 57% of images with potential bias coded as negative from the measles/Disneyland search, 46% of the autism/vaccine images, and 45% of the flu/vaccine images (p = 0.41).

## Discussion

In our study, about half of the images containing medical encounters or vaccines were coded as neutral (i.e. showed a vaccine being administered without showing faces or with needle as the focal point or showed vaccine supplies). Of the remaining photos featuring medical encounters, we found that the likelihood was similar for the use of imagery accompanying online news media related to vaccination containing features which may promote positive or negative sentiments about vaccines. While initially this can be seen as encouraging, as our results do not show a higher proportion of negative images compared to positive imagery, it remains that nearly one in eight photos were coded as negative, containing features which may undermine the public health message or the accurate information about vaccines in the content itself.

The proportions of negative and positive images were not significantly different across search terms which may reflect a random distribution of images in regards to imagery connotation used on news articles. It is quite possible that the inclusion of negative imagery in these news stories is not occurring to intentionally evoke anti-vaccination sentiment, but rather that those choosing the images may not be considering the possible ramifications. The search related to the measles outbreak in Disneyland notably had the lowest percentage of positive and negative imagery, while the search related to flu vaccines had the highest percentage of positive and negative imagery. We surmise that these trends were largely due to relatively high and low proportions of irrelevant images in the measles and flu searches, respectively, and merely reflect the type of news story with the former lending itself to pictures of Disneyland or Mickey Mouse (coded as *irrelevant*), and flu news coverage skewing more medical.

Regardless of intent, however, the prevalence of imagery which may promote negative sentiments about vaccinations raises some concerns. The way in which vaccine-related communication is crafted can have a profound impact on the public’s perception of vaccination’s risk and benefit. A study by Betsch, et. al (2010) found that just a few minutes of browsing on vaccine-critical websites increases perception of risk of vaccines compared to browsing a pro-vaccine site [30]. A follow-up study by the same group also found that even in the face of pro-vaccine statistical information, the negative emotive appeal from a narrative can override the objective information [31].

The ability of images to persuade is particularly noteworthy. A systematic review in 2006 of articles assessing the effects of images in health communication revealed that pictures with text versus text alone have the ability to change adherence to health instructions and other target behaviors based on emotional response [13]. It has been theorized that images provide affect and help to complement textual information [14]. As social psychologist Joffe (2008) states, “without affect, information lacks meaning and will not be used for judgment and decision-making.” (Joffe, 2008, p. 7). For example, when a face is portrayed, the audience is often brought into an emotional state where judgments are made. This emotional connection has been offered to explain why people will feel more compelled to donate to a single identified victim than if they were presented with statistics about a population [32]. Another unique effect of images is the ability to increase information recall compared to text [13]. If a parent more easily remembers the image of multiple needles or a screaming child than the statistics portrayed in an article which highlight the value of vaccination in protection from, say, the reemerging threat of measles, the parent may perceive the screaming child as more memorable, and ultimately more relevant.

In our study, the criteria that defined images as positive or negative were related to the common fears of vaccines, including concerns about the child receiving too many vaccines and overwhelming or eroding the immune system, child cruelty, infringement on parental rights, and general mistrust of the health care system [2, 3]. Needle phobia was also a large contributing factor to the criteria for negative imagery, such as multiple or large needles. According to a survey done in 2011, 7% of over 800 parents cited needle phobia as the primary reason for vaccine non-compliance in their children [33]. Another survey of over 2,000 adults in 2012 showed that 23% would skip the flu vaccine due to fear of needles [34]. Though evidence is lacking on the specific types of needle imagery which may invoke fear, social cognition theory suggests that fear-based stimuli occurs very commonly, such as with phobias of blood and aversion of looking at images containing blood [14]. We chose to categorize images where more than half of the image was taken up by the needle as negative, but future research would need to be done to determine if more or less of the needle showing triggers such anxiety. Images that convey negative sentiments by making the needle the focal point, showing multiple needles, or highlighting the pain can therefore potentially evoke related fears. In contrast, images that provide reconciliation for these fears could evoke positive sentiment from the viewer and potentially promote behaviors that support public health endeavors. For example, pictures of provider-patient dialogue or children smiling during the vaccination or afterwards with a bandage on can counter the mistrust of the health care system or needle phobia. Being aware of these commonly cited fears of vaccines may better equips journalists and editors to avoid or counter stoking those fears through imagery choice, especially when the goal of the editorial content is to provide the most accurate information about vaccinations.

It is possible that editors in the media may feel it is necessary to use an image showing a needle or a person receiving an injection to adequately convey that the article is about vaccination. Yet, articles about urinary tract infections in children, for example, do not need to show catheterization or a urine sample. Moreover, in our sample, images which did not contain any imagery related to vaccination (i.e. coded as irrelevant) made up the majority of photographs used. This underscores that vaccine imagery–positive or negative–need not be used at all in these stories, and this may be a valid strategy for decision-makers covering vaccine news. Vaccination is not alone, of course, in having common iconic default imagery (child receiving injection) in the media; articles about asthma may often show a child using an inhaler, for example. However, whereas asthma is a chronic disease and does not represent a choice of the caretaker, ongoing concerns about public trust surrounding vaccination suggest that greater care and creativity may be worth considering for vaccine stories among those choosing images to accompany news articles.

A separate but related counterargument against avoiding negative imagery in vaccination stories might focus on the need for independence in the press. That is, since a primary goal in health journalism is to convey accurate, balanced information without appearing to have bias or to venture into advocacy [35]–even in favor of well-supported, evidence-based public health measures–editors, reporters, and others creating media for the public may argue that showing a child screaming while receiving an injection is simply an accurate portrayal of vaccination and that it is not their responsibility to specifically choose an image with a positive connotation. This justification, however, presumes that a screaming child receiving an injection is the only possible accurate “reality” of vaccination, which is not the case. Images about birth, for example, may show the result of birth—holding a baby—as opposed to the actual act of the birth itself, and a corollary for vaccination images would be showing the bandage afterward, or the conversation before, as opposed to the act of the injection itself.

Even showing the act of the injection, however, need not appear with negative affect. The breadth of stock and news imagery available for vaccines shows what doctors already know: not every child who receives a vaccine grimaces and screams. It is possible that some editors beholden to the popular journalistic axiom “if it bleeds, it leads” may choose more literal vaccination images based on their dramatic effect, without recognizing the potential public health risks of negative affect in those images. Our hope is that promoting awareness of the potential public health impact may lead to more deliberate photo selections that still “show” the act of vaccination without explicitly including the negative affect. Ongoing concerns about public trust surrounding vaccination suggest that greater care and creativity may be worth considering for vaccine stories among those choosing images to accompany news articles.

Our study has several limitations. While Google is currently the most popular search engine, it is possible that using a single search tool may have limited what news results were generated and thus analyzed. Similarly, while we chose to search terms aimed at capturing a variety of news stories about vaccination, these representations were assumed and not empirically tested. Additionally, we did not analyze the connotation of the news articles themselves and whether the connotation of the associated imagery matched. This may have shed light on the frequency of negative or positive articles and provided some context for the images. However, we sought to analyze the connotation of the images at face value as imagery alone has been shown to impact the reader’s interpretation of the accompanying news. Moreover, our primary goal was less to establish a firm frequency of the use of negative versus positive images that would apply across all vaccine news coverage, but rather to generate a sample that highlights that there is in fact a difference, and that stakeholders who make a choice in image selection can have a framework to consider. Lastly, the classification criteria used in this study deemed certain images as being associated with a particular sentiment though these associations were assumed as little empirical research has been done to support these associations.

## Conclusion

In light of the increasing prevalence of vaccine hesitancy, our study brings new theoretical and practical information to consider when crafting health communications about vaccines. As journalists play an essential role in public health, we hope that with this new information, editors and writers of news covering vaccine-related topics can be better informed about selecting images that support public health initiatives regarding vaccines when appropriate. Our data suggests that there still exists a great need for images that communicate encouraging information about vaccines and the protection it provides for our children as nearly one in eight images contained features which may promote negative sentiments such as fear or mistrust about vaccination.
